# The Effects of Disease‐Modifying Therapies on Optic Nerve Degeneration in Multiple Sclerosis

**DOI:** 10.1111/ene.70081

**Published:** 2025-03-06

**Authors:** Xia Zhang, Shuang Song, Bo Chen, Letizia Leocani, Xinyu Zhao, Yong Zhong, Marco Pisa, Srilakshmi M. Sharma

**Affiliations:** ^1^ Department of Ophthalmology Peking Union Medical College Hospital, Chinese Academy of Medical Sciences and Peking Union Medical College Beijing China; ^2^ Department of Ophthalmology Oxford University Hospitals NHS Trust Oxford UK; ^3^ Department of Biostatistics, Harvard T.H. Chan School of Public Health Harvard University Boston Massachusetts USA; ^4^ Department of Neurology, Tongji Hospital of Tongji Medical College Huazhong University of Science and Technology Wuhan China; ^5^ Nuffield Department of Clinical Neuroscience University of Oxford Oxford UK; ^6^ Neurological Department and Institute of Experimental Neurology (INSPE) Scientific Institute, Hospital San Raffaele Milan Italy

**Keywords:** disease‐modifying therapy, ganglion cell layer, multiple sclerosis, optic coherence tomography, retinal nerve fiber layer

## Abstract

**Background:**

Retinal nerve fiber layer (RNFL) and ganglion cell‐inner plexiform layer (GCIPL) thinning are used as markers of subclinical retinal degeneration to evaluate the effect of disease‐modifying therapies (DMTs) on disease progression in clinical trials of multiple sclerosis (MS). This study aimed to assess the available evidence regarding the effects of DMTs on retinal thinning in people with MS.

**Methods:**

Databases were searched for studies reporting longitudinal optical coherence tomography (OCT)‐derived annualized RNFL and GCIPL thinning in patients receiving DMTs treatment. The standardized mean differences (Hedges g) of RNFL and GCIPL thickness between the baseline and follow‐up were used as the primary effect size measure. DMTs were divided into moderate (M‐DMTs) and high (H‐DMTs) efficacy therapies.

**Results:**

Twenty‐one studies including 2158 patients and 3685 eyes were included. Overall, significant annualized RNFL (*g* = −0.6715, *p =* 0.0077) and GCIPL (*g* = −0.31, *p* < 0.0001) thinning was observed at follow‐up compared with baseline. Annualized RNFL thinning was only significant in the M‐DMTs group (*g* = −0.6992, *p* = 0.0243). Annualized GCIPL thinning was significant in both M‐DMTs (*g* = −0.38, *p =* 0.0006) and H‐DMTs group (*g* = −0.19, *p* < 0.0001) but was significantly greater in the M‐DMTs group compared with the H‐DMTs group (*g* = −0.20, *p =* 0.0017). There was no difference in annualized GCIPL or RNFL thinning between RRMS and PMS, or between RRMS with and without ON history.

**Conclusions:**

High‐DMTs are more effective in reducing longitudinal thinning of RNFL and GCIPL compared with M‐DMTs. GCIPL thinning could serve as a sensitive predictor for the surveillance of optic nerve degeneration and the assessment of DMT efficacy for both RRMS and PMS.

## 1 Introduction

Multiple sclerosis (MS) is the most prevalent chronic inflammatory demyelinating disease of the central nervous system (CNS). Neurodegeneration is the main determinant for long‐term disability among people with MS (PwMS). Progressive neuro‐axonal loss is detectable from the earliest phases, but reliable measures to monitor neurodegeneration are lacking [1]. Neurodegeneration along the visual pathway correlates strongly with disease progression and neurodegeneration in the CNS, [2, 3] can be detected by optical coherence tomography (OCT), and is characterized by the loss of retinal ganglion cells (RGCs) and their axons (the retinal nerve fiber layer, RNFL). It is well established that PwMS display a progressive thinning of the RNFL and ganglion cell‐inner plexiform layer (GCIPL), even without a history of optic neuritis (ON) [4]. The degree of retinal thinning is strongly associated with greater brain atrophy, [5, 6] higher levels of visual [7] and global disability, [8] and lower quality of life [9]. Therefore, these OCT measures can serve as sensitive indicators for monitoring disease progression and assessing the efficacy of putative neuroprotective drugs.

Disease‐modifying therapies (DMTs) are the standard treatment for PwMS [10] and can be divided into high‐efficacy therapies (H‐DMTs), such as ocrelizumab, natalizumab, and alemtuzumab, and moderate efficacy therapies(M‐DMTs), such as beta‐interferon and glatiramer acetate [11]. Their efficacy is commonly measured by the relapse rate, overall disability (e.g., EDSS score), and MRI‐derived metrics (e.g., lesion activity, burden, or brain volume), [12, 13, 14] which may underestimate functional and structural impairment of the optic nerve [15]. Given the sensitivity of OCT‐derived RNFL and GCIPL loss in detecting subclinical progression, these measures have been increasingly utilized as outcomes to evaluate the efficacy of various DMTs in clinical trials [16, 17, 18]. However, there is no consensus regarding the effectiveness of DMTs on OCT‐derived outcomes, nor has there been a comparison of H‐DMTs with M‐DMTs in this context. Thus, this study aimed to conduct a systematic review and meta‐analysis of the available evidence regarding the effects of DMTs on OCT‐derived metrics of optic nerve degeneration in MS.

## 2 Method

### 2.1 Protocol and Registration

This study was performed in accordance with the Preferred Reporting Items for Systematic Reviews and Meta‐analyses (PRISMA) statement [19] and adhered to the Tenets of the Declaration of Helsinki.

### 2.2 Eligibility Criteria

The inclusion criteria were as follows: (1) enrollment of PwMS diagnosed according to the latest diagnostic criteria at the time of study publication; (2) enrollment of people aged ≥ 18 years; (3) availability of longitudinal OCT data on DMT treatment; (3) availability of either: (a) RNFL or GCIPL thickness values at baseline and at ≥ 1 year follow‐up visit; (b) baseline RNFL or GCIPL thickness and annualized or overall RNFL or GCIPL thickness change at ≥ 1 year follow‐up; (4) OCT metrics reported as means and standard deviations (SDs) or in a comparable format (e.g., *z* values, standard errors [SE], 95% confidence intervals [CI]) in accordance with the Preferred Reporting Items for Systematic Reviews and Meta‐Analysis [19]; and (5) full text available.

We excluded studies that included: (1) eyes with ON or corticosteroid treatment within 30 days prior to enrolment or during follow‐up; (2) subjects with ocular or systemic comorbidities that would affect the quality or accuracy of OCT data; (3) case–control discrepancies, other than DMT administration, that would introduce significant bias (confirmed by two researchers X.Z. and M.P.); (4) OCT devices other than the Spectralis (Heidelberg Engineering GmbH, Heidelberg, Germany) or Cirrus/Stratus (Carl Zeiss Meditec Inc., Dublin, CA) or studies that failed to specify device information upon request. We also excluded: (5) studies that did not clarify the inclusion of acute ON eyes in their analyses or did not identify subjects' MS subtypes (relapsing–remitting MS [RRMS], secondary progressive MS [SPMS], and primary progressive MS [PPMS]); (6) studies with insufficient data for estimating a relative risk or weighted mean difference (WMD), as confirmed by two researchers (X.Z. and M.P.); (8) If RCTs or cohort studies shared the same affiliations or authors, the current researchers contacted the corresponding author to determine whether the study was a duplicate. Studies confirmed to be duplicates or studies by corresponding authors who did not respond within 3 months were excluded; (9) Literature reviews, editorials, conference abstracts or technical notes.

### 2.3 Information Sources and Search Strategy

The Ovid Medline (OvidSP), Ovid Embase (OvidSP), PubMed, Web of Science, CINAHL, and Ovid databases were searched from January 1990 to April 2023 without restrictions regarding language or the publication date. The DMTs included in the systematic search were grouped into high‐efficacy DMTs (fingolimod, siponimod, ponesimod, ozanimod, laquinimod, natalizumab, alemtuzumab, daclizumab, ocrelizumab, cladribine, rituximab, and ofatumumab) and moderate‐efficacy DMTs (β‐interferon, peginterferon, glatiramer acetate, mitoxantrone, azathioprine, cyclophosphamide, teriflunomide, and dimethyl fumarate). The full search strategy is provided in Data S1. The reference lists of all included studies and conference abstracts from the European Committee for Treatment and Research in Multiple Sclerosis (ECTRIMS) and the Americas Committee for Treatment and Research in Multiple Sclerosis (ACTRIMS) conferences were manually searched to identify additional related studies. All searches were reconducted before the final analysis. Data selection and extraction strategy was demonstrated in Data S2.

### 2.4 Statistical Analysis

The metaphor package [20] R version 4.3.2 (R Foundation for Statistical Computing, Vienna, Austria) was used to calculate the main summary effect size, representing differences between baseline and follow‐up RNFL and GCIPL thickness, and it was divided by the pooled SD for their respective outcomes, as previously described by Landmeyer et al. [21]. Negative effect sizes indicated optic nerve degeneration. Moderation analyses were conducted to assess the potential influences of clinical and demographic variables. The potential moderators were identified a priori and included the time interval from the active phase, disease duration, follow‐up time, baseline EDSS score, baseline RNFL/GCILP thickness, sex, age, and study quality score. Publication bias was investigated by visual analysis of funnel plots, Egger's regression test, and the trim‐and‐fill method [22].

## 3 Results

### 3.1 Results of the Systematic Review

**FIGURE 1:**
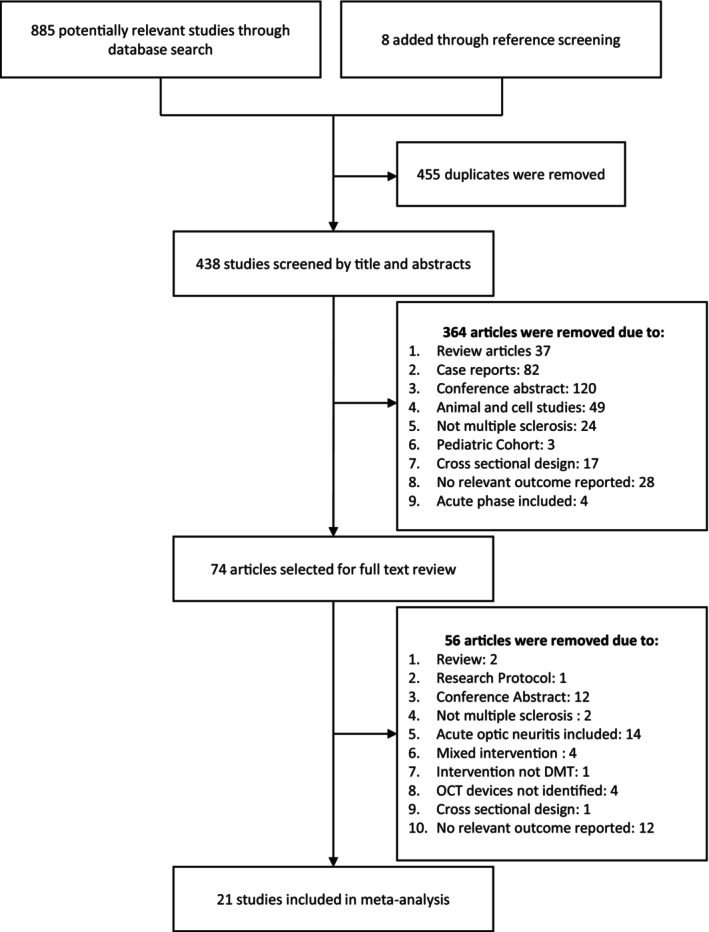
Flowchart illustrating the screening and study selection process for the systematic review and meta‐analysis.

We initially identified 893 potentially relevant studies. After excluding duplicates and screening the titles and abstracts, the full texts of 74 articles were reviewed. Ultimately, 16 studies met the inclusion criteria. Additionally, five articles that met the inclusion criteria but that did not report outcomes stratified by the DMT group were included in subgroup analyses. Therefore, 21 studies comprising 2158 patients (3685 eyes) were included in the present systematic review and meta‐analysis. The PRISMA flowchart describing the screening process is shown in Figure 1, and the main characteristics of the included studies are reported in Table S1.

Four of the included studies were randomized controlled trials (RCTs), one was an open‐label clinical trial, 12 were prospective observational studies, and four studies were retrospective.

### 3.2 Distribution of Treatments

Table 1 shows the distribution of DMTs among included studies. Longitudinal OCT data from people with MS receiving moderate efficacy therapies were investigated in 12 independent studies (*k* = 13 samples); data on high‐efficacy therapies were reported in 13 studies (*k* = 14); and placebo/DMT‐naïve cohorts (*k* = 6) were reported in six studies. The most commonly studied DMT was β‐interferon (*k* = 12), followed by glatiramer acetate (*k* = 11), natalizumab (*k* = 9), and fingolimod (*k* = 8). No articles reported longitudinal OCT outcomes for mitoxantrone, cyclophosphamide, laquinimod, siponimod, ponesimod, ozanimod, or ofatumumab.

**TABLE 1:** Number of patient samples reported and total number of patients grouped by treatment.

Treatment	Samples
Moderate efficacy therapies	13^a^
*Interferon‐beta*	12
*Glatiramer Acetate*	11
*Dimethylfumarate*	2
*Teriflumomide*	2
*Azathioprine*	1
High‐efficacy therapies	14
*Natalizumab*	9
*Fingolimod*	8
*Alemtuzumab*	3
*Rituximab*	2
*Ocrelizumab*	1
*Daclizumab*	1
*Acrelizumab*	1
*Cladribine*	1

^a^ The total number of samples exceeds the reported number for both moderate efficacy therapies (13 samples) and high‐efficacy therapies (14 samples) because some patient samples were counted in more than one treatment category. Details of these overlapping samples are provided in Table S1.

### 3.3 Results of the Meta‐Analysis

#### 3.3.1 Patient Group Characteristics

Table 2 shows the demographic and clinical characteristics of the combined cohort from all 21 studies (2158 patients, 3685 eyes) and of the subgroups of people with MS receiving M‐DMTs and H‐DMTs. The mean age of the entire cohort was 42.26 (± 9.69) years. The proportion of females was 70.28% (± 10.96). The proportion of eyes with a history of ON was 35.57% (± 11.96). The mean follow‐up duration was 2.98 (± 1.90). The mean disease duration was 9.90 (± 7.80) years. The mean EDSS score at baseline was 2.62 (± 1.55). The clinical characteristics of the M‐DMTs and H‐DMTs groups are also presented in Table 2. The clinical characteristics of the M‐DMTs and H‐DMTs groups are also presented in Table 2. The H‐DMT group has a lower proportion of ON history, a higher EDSS score, thinner RNFL thickness, and thicker GCIPL thickness. Notably, the entire cohort had a higher EDSS score than the M‐DMTs and H‐DMTs groups; this is due to the inclusion of people with PMS (PwPMS) in subgroup studies.

**TABLE 2:** Demographics of the full patient sample included in the meta‐analysis, stratified by type of treatment.

	Overall^a^ *n* = 2158 (3685 eyes)	M‐DMTs^b^ *n* = 460 (912 eyes)	H‐DMTs^b^ *n* = 503 (1427 eyes)	N‐DMTs *n* = 467 (792 eyes)	*p* ^c^
Age(yr ± SD)	42.26 ± 9.69	38.73 ± 9.51	39.04 ± 9.41	44.72 ± 8.35	0.612
Female (%)	70.28 ± 10.96 (SE)	75.45 ± 14.67 (SE)	74.88 ± 13.79 (SE)	67.46 ± 27.06 (SE)	0.534
ON history (%)	35.57 ± 11.96 (SE)	35.12 ± 17.81 (SE)	31.86 ± 15.56 (SE)	31.86 ± 26.95 (SE)	**3.22e‐06**
Follow‐up time (yr ± SD)	2.98 ± 1.90	2.63 ± 3.28	2.37 ± 1.60	2.22 ± 0.31	0.113
Disease duration (yr ± SD)	9.90 ± 7.80	7.48 ± 6.91	8.77 ± 7.05	9.25 ± 7.93	0.612
Baseline EDSS score^a^ (point±SD)	2.62 ± 1.55 (including PPMS)	1.84 ± 1.41	2.02 ± 1.35	2.95 ± 1.08	**0.043**
Baseline RNFL (um ± SD)	91.99 ± 14.17	98.84 ± 15.0	89.71 ± 13.90	92.14 ± 13.68	**9.58e‐49**
Baseline GCIPL (um ± SD)	76.92 ± 10.64	74.59 ± 10.59	78.16 ± 10.66	—	**3.64e‐15**
Pooled RNFL ANL (95% CI)	**−0.6715 (−1.14, −0.20)**	**−0.6992 (−1.30, −0.10)**	−0.6249 (−1.40, 0.15)	−0.46 (−0.61, −0.31)	0.87^c^
Pooled GCIPL ANL (95% CI)	**−0.3072 (−0.41, −0.20)**	**−0.3816 (−0.44, −0.32)**	**−0.1860 (−0.28, −0.09)**	—	**0**.**0017** ^c^

*Note:* Bold font represent *p* value < 0.05; *p* value of pooled annualized thinning of RNFL thickness is 0.0077 (overall), 0.0243 (M‐DMTs), 0.1062 (H‐DMTs); *p* value of pooled annualized thinning of GCIPL is < 0.0001 (overall), 0.0006 (M‐DMTs), < 0.0001(H‐DMTs).

Abbreviations: ANL, annualized loss; CI, confidence interval; DMT, disease‐modifying therapies; EDSS, Expanded Disability Status Scale; GCIPL, ganglion cell‐inner plexiform layer; H‐DMT, high‐efficacy DMTs; M‐DMTs, moderate efficacy DMTs; N‐DMTs, placebo‐treated/no‐DMT‐treated; OCT, optic coherence tomography; ON, optic neuritis; PPMS, primary progressive multiple sclerosis; RNFL, retinal nerve fiber layer; SD, standard deviation; SE, standard error.

^a^ Overall cohorts include progressive multiple sclerosis (*n* = 587) and studies did not clarify H‐DMT/M‐DMT types (*n* = 141). The overall cohort had a higher EDSS score than the M‐DMTs and H‐DMTs groups.

^b^ Sotirchos and Wang's cohort did not present patient numbers in each group and was only included in the sum of eye numbers.

^c^ *p* Value column presented comparison between M‐DMTs cohorts and H‐DMTs cohorts.

#### 3.3.2 Treatment Effects Between Baseline and Follow‐Up

**FIGURE 2:**
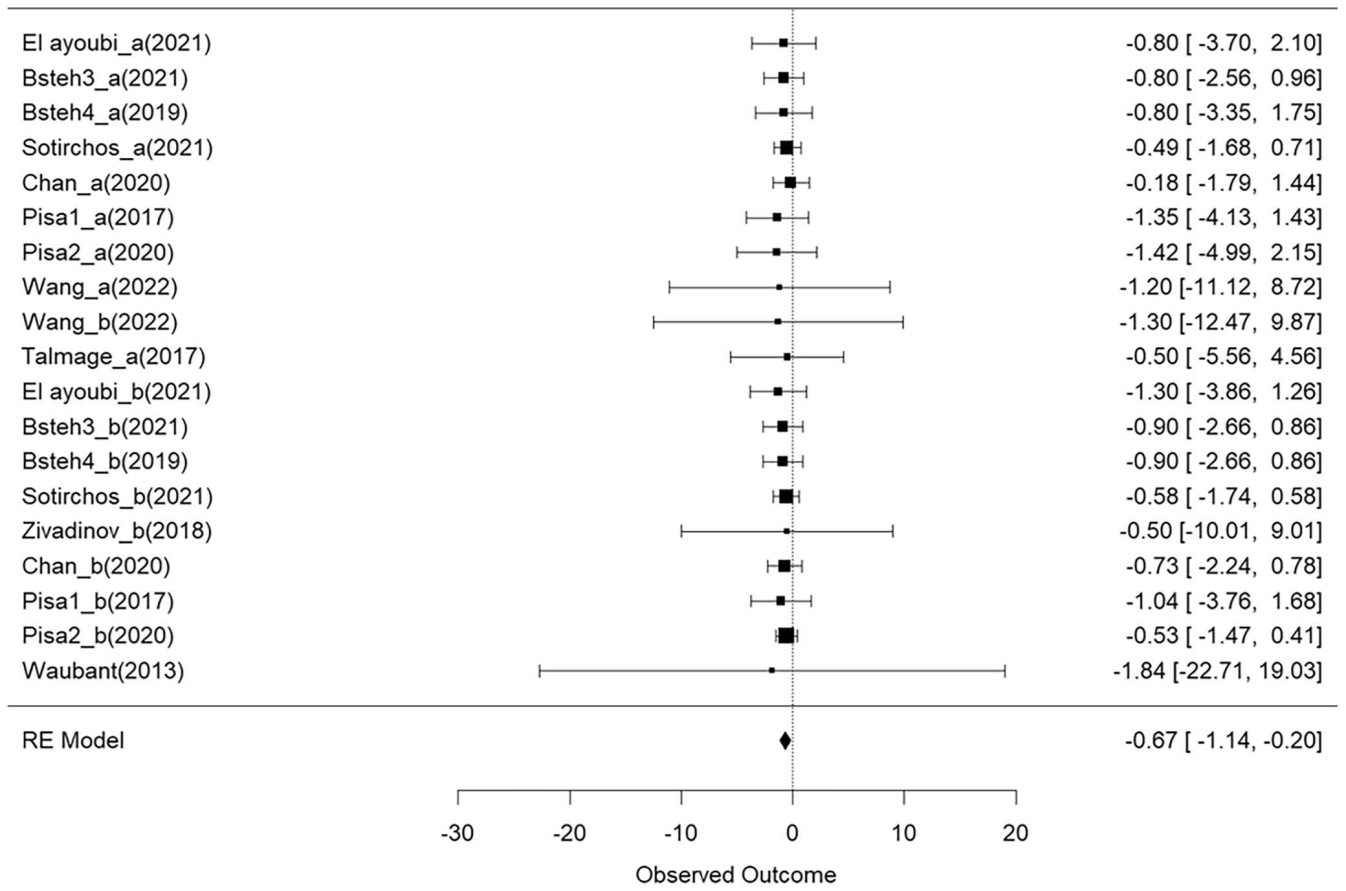
Forest plot visualizing the mean effect sizes of longitudinal decreases in RNFL thickness in different study cohorts. Effect sizes are presented as Hedges g. Negative effect sizes indicate a decrease in RNFL thickness between baseline and follow‐up. Confidence intervals crossing the zero line indicate no significant decrease in RNFL thickness in the cohort.

First, we detected whether the annualized thinning of RNFL or GCIPL is significant in M‐DMTs and H‐DMTs treated population by comparing the baseline thickness and follow‐up thickness. Overall, a significant annualized RNFL thinning was observed between baseline and follow‐up OCT assessments across the study included, with an effect size of −0.6715 (95% CI = [−1.14 to −0.20], *p =* 0.0077) (Figure 2). Specifically, the M‐DMT group showed a significant decrease in RNFL thickness between baseline and follow‐up, with a small to moderate effect size (gM‐DMTs = −0.6992, 95% CI = [−1.30 to −0.10], *p =* 0.0243), while no significant decrease in RNFL thickness was detected in the H‐DMT group (gH‐DMTs = −0.6249, 95% CI = [−1.40–0.15], *p =* 0.1062). However, the difference in annualized RNFL thinning between the H‐DMTs and M‐DMTs cohorts did not reach statistical significance (*p =* 0.87). The SD between outcomes (τo) was < 0.0001, indicating minimal variance attributable to heterogeneity across the included studies.

**FIGURE 3:**
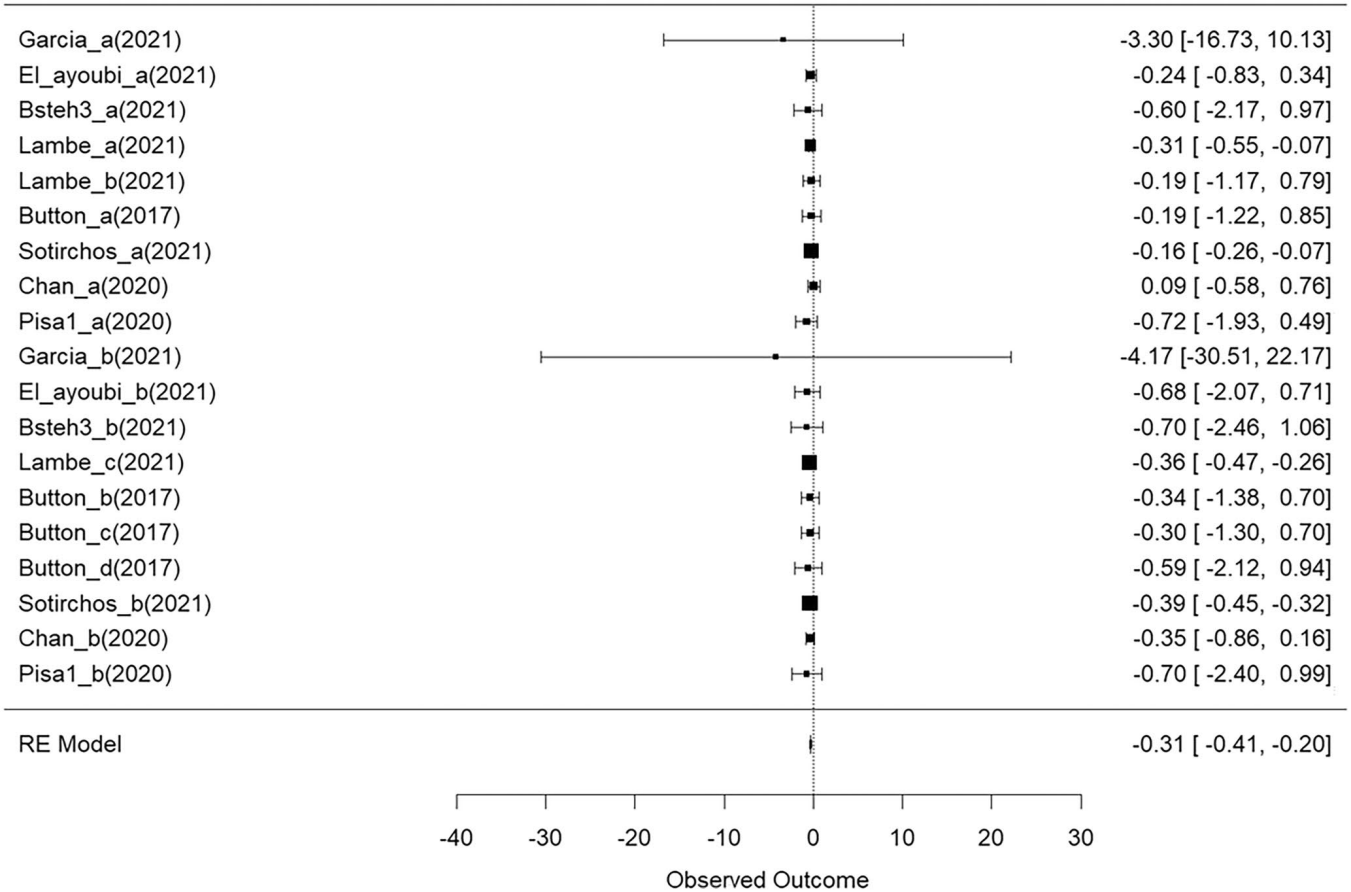
Forest plot visualizing the mean effect sizes of longitudinal decreases in GCIPL thickness in different study cohorts. Effect sizes are presented as Hedges g. Negative effect sizes indicate a decrease in GCIPL thickness between baseline and follow‐up. Confidence intervals crossing the zero line indicate no significant decrease in GCIPL thickness in the cohort.

Significant annualized GCIPL thinning was observed in the overall cohort (goverall = −0.3072, 95% CI [−0.41, −0.20], *p* < 0.0001) (Figure 3). GCIPL thinning was confirmed in both the H‐DMTs and M‐DMTs cohorts, with small‐to‐moderate effect sizes (gM‐DMTs = −0.3816, 95% CI = [−0.44 to −0.32], *p =* 0.0006; gH‐DMTs = −0.1860, 95% CI = [−0.44 to −0.32], *p* < 0.0001). Importantly, annualized thinning of GCIPL thickness was greater in the M‐DMTs group compared with the H‐DMTs group, indicating that H‐DMTs were more effective than M‐DMTs therapies in terms of preventing the thinning of GCIPL thickness (gM‐DMTs vs. H‐DMTs = −0.20, 95% CI = [−0.31 to −0.09], *p =* 0.0017). The SD between outcomes (τo) was < 0.0001, indicating an inconsequential degree of variance attributable to heterogeneity across the included studies.

#### 3.3.3 Subgroup Analysis

**FIGURE 4:**
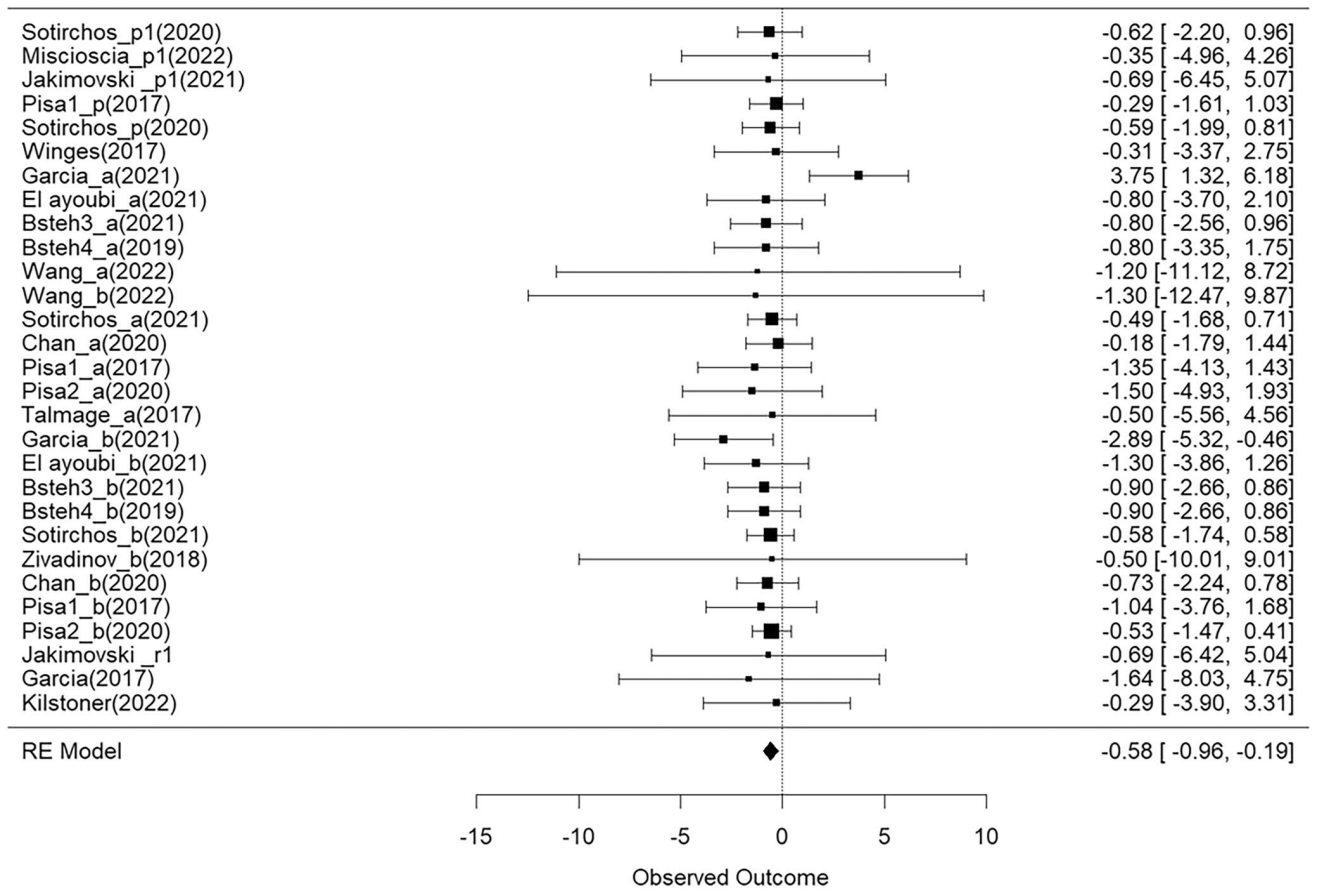
Forest plot visualizing the mean effect sizes of longitudinal decreases in RNFL thickness in RRMS and PMS subgroup studies. The top six samples (Sotirchos_p1, Miscioscia_p1, Jakimovski_p1, Pisa_p, Sotirchos_p, Winges) represent PMS cohorts. Effect sizes are presented as Hedges g. Negative effect sizes indicate a decrease in RNFL thickness between baseline and follow‐up. Confidence intervals crossing the zero line indicate no significant decrease in RNFL thickness in the cohort.

A subgroup analysis focusing on RNFL thinning in progressive multiple sclerosis (PMS) was run on data from six independent studies. No significant RNFL change was observed in the PMS cohort (gPMS = −0.47, 95% CI = [−1.28, 0.34], *p* = 0.24), and no significant difference in annualized RNFL thinning between the PMS and RRMS cohorts was observed (Figure 4). Analysis of GCIPL between MS subtypes was not possible due to the limited number of studies available (*n* = 3).

Subgroup analysis comparing eyes with vs. without a history of ON (RRMS‐ON and RRMS‐NON, respectively) was completed considering 21 published cohorts. No significant decrease in RNFL thickness was detected in the RRMS‐ON (gRRMSON = −0.92, 95% CI = [−2.24, 0.41], *p =* 0.16) or the RRMS‐NON group (gRRMSNON = −0.55, 95% CI = [−1.49, 0.39], *p =* 0.24), and there was no difference detected between the two cohorts (*p =* 0.64).

**FIGURE 5:**
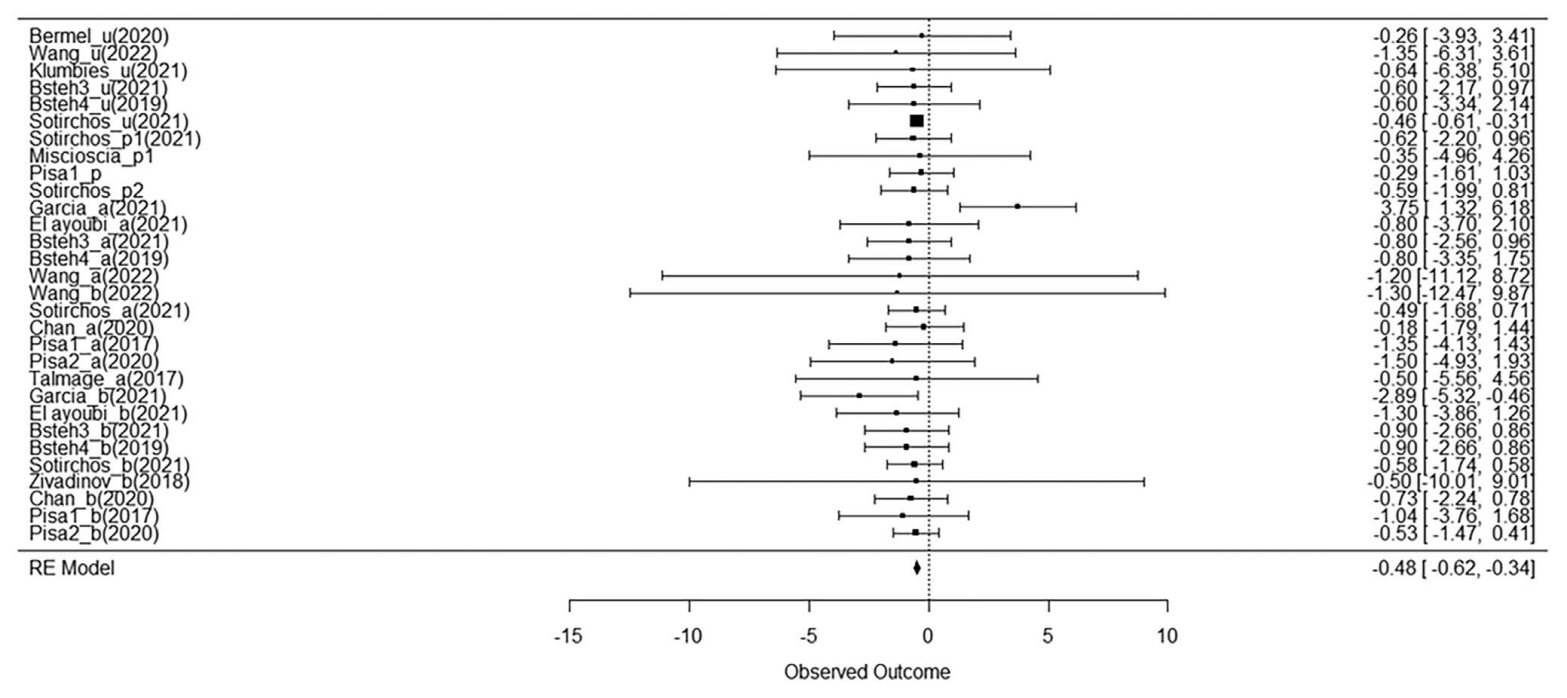
Forest plot visualizing the mean effect sizes of longitudinal decreases in RNFL thickness in placebo‐treated/untreated cohorts. The top six samples (Bermal_u, Wang_u, Klumbies_u, Bsteh3_u, Bsteh4_u, Sotirchos_u) represent placebo‐treated/untreated cohorts. Effect sizes are presented as Hedges g. Negative effect sizes indicate a decrease in RNFL thickness between baseline and follow‐up. Confidence intervals crossing the zero line indicate no significant decrease in RNFL thickness in the cohort.

Finally, six studies reported RNFL thickness changes in DMT‐naïve (N‐DMT) or placebo‐treated cohorts, with three of which including people with RRMS (PwRRMS) and three people with PMS (PwPMS). Therefore, the PMS and RRMS cohorts were pooled to evaluate the difference in annualized RNFL thinning between the untreated/placebo group and the DMT‐treated group. Four cohorts of PwPMS and 20 cohorts of PwRRMS were included in the DMT‐treated group. The results revealed a significant decrease in RNFL thickness in both the untreated/placebo group (gNDMT = −0.46, 95% CI = [−0.61, −0.31], *p* < 0.001) and the DMT‐treated group (gMIXEDDMT = −0.56, 95% CI = [−0.97, −0.18], *p =* 0.005), with no substantial difference detected between the two groups (*p =* 0.5783). The N‐DMT group was further compared with the H‐DMTs group, and no significant differences were detected between the two groups (*p =* 0.6697, Figure 5). Notably, only two cohorts in the H‐DMTs group included PwPMS.

#### 3.3.4 Moderation Analysis

Moderation analyses were conducted to examine the influence of potentially confounding covariates, including sex, age, time to active phase, follow‐up duration, disease duration, baseline EDSS score, baseline RNFL thickness, OCT device, and study quality. None of the potential moderators significantly affected the annualized thinning of RNFL or GCIPL thickness (Table 3).

**TABLE 3:** The effect of covariates on the responsiveness of RNFL and GCIPL to DMTs.

Outcomes	RNFL				GCIPL			
Moderator	Estimate	95% CI	*p*	*k*	Estimate	95% CI	*p*	*k*
Age	0.03	−0.10 to 0.16	0.60	19	−0.005	−0.05 to 0.04	0.79	17
Female	0.49	−4.29 to 5.27	0.83	19	1.06	−1.09 to 3.21	0.31	19
ON history	0.08	−4.52 to 4.69	0.97	17	0.87	−0.63 to 2.37	0.23	19
Time to active phase	0.09	−0.45 to 0.63	0.73	19	0.02	−0.02 to 0.06	0.30	19
Follow‐up time	0.10	−0.40 to 0.60	0.68	19	0.06	−0.10 to 0.23	0.45	19
Disease duration	0.01	−0.27 to 0.28	0.95	19	−0.02	−0.06 to 0.02	0.33	17
Baseline EDSS score	0.13	−0.82 to 1.08	0.77	19	0.0013	−0.45 to 0.45	0.99	12
Baseline RNFL thickness	0.001	−0.13 to 0.13	0.99	19	—	—	—	—
Baseline GCIPL thickness	—	—	—	—	−0.02	−0.18 to 0.15	0.85	18
OCT device‐Heidelberg	−0.07	−1.08 to 0.94	0.88	19	−0.10	−0.31 to 0.11	0.32	19
OCT device‐stratus	−0.69	−8.29 to 6.91	0.85	19	—	—	—	—
Study quality	−0.0005	−0.17 to 0.17	0.99	19	0.003	−0.03 to 0.05	0.86	19

*Note:* k = number of populations in which the respective moderator variable was reported.

Abbreviations: CI, confidence interval; DMT, disease‐modifying therapies; EDSS, Expanded Disability Status Scale; GCIPL, ganglion cell‐inner plexiform layer; OCT, optic coherence tomography; ON, optic neuritis; RNFL, retinal nerve fiber layer.

#### 3.3.5 Sensitivity Analysis

We included cohorts with baseline examinations more than 1 months after ON attack. However, we were aware that one‐month interval may be too short for full resolution of ON‐induced changes and the ON‐induced RNFL and retinal atrophy is possibly still ongoing. Thus, we conducted a sensitivity analysis by excluding the cohorts with OCT testing within 3 months post‐ON attack (Talmage, Wang, Zivadinov, Waubant, Table S1), which confirmed the same findings of our primary outcome (Figure S1).

## 4 Discussion

To our knowledge, this is the first systematic review and meta‐analysis that specifically gathers and evaluates the available evidence regarding the effects of DMTs on OCT‐derived metrics. This meta‐analysis demonstrates the presence of a significant annualized RNFL and GCIPL thinning in PwMS and suggests that H‐DMTs are more effective in preventing neurodegeneration along the visual pathway compared to M‐DMTs. Furthermore, our data support the hypothesis that GCIPL is a more sensitive measure than RNFL to monitor neurodegeneration using OCT.

OCT, a non‐invasive technique utilizing near‐infrared light to assess the thickness of retinal tissues, [23] has been increasingly recognized as a pivotal instrument for monitoring individuals with MS. The advent of spectral domain OCT (SD‐OCT) has enhanced resolution capabilities, achieving measurements as precise as 2–3 μm in retina thickness. This contrasts markedly with the 1.0*1.0*1.2‐mm resolution offered by MRI for detecting brain atrophy in PwMS [24]. Notably, OCT has demonstrated reliable and reproducible results in identifying retinal thinning associated with MS across different devices and diverse populations, making it valuable for multicenter clinical trials and observational studies [25, 26].

In terms of validity, OCT has proven to be sensitive in detecting disease activity and progression in MS. Clinical and radiographic evidence of disease activity correlates with greater RNFL [27] and GCIPL thinning [28]. These associations can extend to changes in visual disability, motor and cognitive disability. Several studies have indicated that RNFL and GCIPL thickness correlate with low contrast visual acuity [29] and visual‐specific quality of life [30] in PwMS. Furthermore, RNFL and GCIPL thicknesses are associated with white matter atrophy, cortical thinning, [2] brain atrophy, [31] white matter lesions, [32] and spinal cord thinning [33]. These findings underscore the role of OCT in the evaluation of global disease burden, including the EDSS, [3] thereby highlighting its significance in the surveillance of MS‐related progression.

Responsiveness to therapeutic interventions is a fundamental criterion for validating any new biomarker. Despite the promising utility of OCT in monitoring MS, the responsiveness of OCT findings to DMT has yet to be fully elucidated. Our meta‐analysis identified only three studies examining the differential impacts of treatments (Garcia, [23] Bsteh, [31] and El Ayoubi [24]), underscoring a significant gap in the literature and further substantiating the potential of OCT as a biomarker for treatment response in MS.

The pooled annualized RNFL thinning rate in the H‐and M‐DMT‐treated cohorts in our meta‐analysis was −0.6715 μm/year. Studies before 2014 reported thinning rates ranging from −1.49 μm/year to −1.27 μm/year, [34, 35, 36] while more recent studies report a much lower thinning rate in mixed DMT‐treated cohorts—between −0.36 and − 0.53, [37] which are consistent with our results (which were derived from more recent studies). While this discrepancy may be due to the inclusion of untreated cohorts, mixed MS phenotypes and older OCT devices, it may also indicate that the newly developed DMTs may be more effective in delaying the thinning of the RNFL and subsequently the disease progression.

Our primary meta‐analysis was restricted to PwRRMS actively receiving at least one DMT; therefore, the results cannot be extrapolated to untreated cohorts. To address this, we expanded our analysis to include six cohorts (three RRMS cohorts and three PMS cohorts) comprising patients who were either untreated or receiving placebo in controlled trials. We found that the pooled RNFL thinning rate among these six cohorts was −0.46 μm/year. Additional research into OCT thinning in MS cohorts without DMT treatment may allow us to explore the influence of clinical and demographic variables on retinal thinning rates in PwMS.

We also investigated the differential effects of H‐DMTs versus M‐DMTs on annualized RNFL and GCIPL thinning rates. Significant annualized GCIPL thinning rate was detected in both treatment groups from baseline to the 1‐year follow‐up, with greater annualized thinning rate in the M‐DMTs group. Similarly, significant annualized RNFL thinning was observed only in people on M‐DMTs.

Our findings suggest that GCIPL thinning rate is a more sensitive biomarker for evaluating optic nerve damage in MS than RNFL thickness, corroborating findings from previous investigations. In previous studies, GCIPL thickness was found to have a stronger correlation with low contrast letter acuity in MS than pRNFL thickness [38]. Moreover, GCIPL asymmetries were found to have greater accuracy than RNFL for detecting subclinical optic nerve involvement in CIS [39]. Additionally, reductions in GCIPL thickness have been detected in PwMS without corresponding pRNFL thinning [6]. The superior performance of GCIPL over pRNFL may be attributed to the methodological approach of measuring macular GCIPL thickness, as proposed by IMSVISUAL. This protocol, which employs Spectralis or Cirrus OCT, calculates the average thickness across a macular volume cube (either 200*200 or 512 *128 scans within a 6*6‐mm area), in contrast to RNFL thickness measurements, which are derived from a single 12° circle scan around the optic disc. Furthermore, compared with RNFL measurements, GCIPL measurements have been shown to have higher intraclass correlation coefficients (ICCs) and coefficient reliability (CR) [40].

In our subgroup analyses, the annualized RNFL thinning rates in PwPMS were not significantly different from those in PwRRMS. Additionally, a significant annualized RNFL thinning rate was not detected within the PMS group. We found very few studies which reported longitudinal changes in RNFL thinning in PwPMS, potentially leading to large variances, which may have led to the lack of significant RFNL thinning in the PwPMS group. Nevertheless, within the six studies included, two reported RNFL thinning rates in both PMS and PwRRMS, with both identifying a faster thinning rate of RNFL thickness in PwPMS [41, 42]. To date, few H‐DMTs (ocrelizumab, siponimod) and M‐DMT options (interferon beta‐1b) are available for PwPMS. Given that we have found that the responsiveness of RNFL thickness to different DMTs differs among PwRRMS, more attention should be devoted to the optic structural changes in PwPMS receiving treatment.

Additionally, we did not observe a significant annualized thinning of RNFL thickness in either the RRMSON or RRMSNON cohort. However, the pooled annualized RNFL thinning rate in the ON group was higher compared with the NON group. In vitro studies have suggested that chronically demyelinated axons are more susceptible to neurodegeneration than those that have undergone complete remyelination. However, previous OCT studies have reported conflicting results regarding this hypothesis. Pisa et al. [27] observed no significant difference in longitudinal RNFL thinning between eyes with abnormal or normal visual evoked potentials (VEP) in PwMS. Conversely, Klistorner et al. [43] reported a significantly greater rate of RNFL thinning in ON eyes compared with the fellow eye. Our meta‐analysis revealed a greater, though nonsignificant, RNFL thinning rate in the ON cohort compared with the non‐ON cohort, potentially indicating a mild effect of chronic demyelination on RNFL thinning. In addition, arms with history of optic neuritis, especially with multiple relapses were more likely to have a significantly thinner RNFL layer than nonoptic neuritis PwMS, [39] which may influence OCT segmentation accuracy [44].

Unlike the non‐DMT/placebo‐treated patients, we did not detect a significant annualized thinning in RNFL thickness in H‐DMT‐treated patients, which suggests that H‐DMTs may contribute to the protection against optic nerve degeneration. Nevertheless, we did not find a significant difference in the rate of RNFL thinning between the Mixed‐DMT and untreated/placebo groups, nor between the H‐DMT and untreated/placebo groups. However, we may have been underpowered in detecting a difference in RNFL thinning rates with N‐DMT, as only six placebo/untreated PwMS cohorts were available in the literature for this meta‐analysis. Meanwhile, these N‐DMT cohorts included people with MS with different disease subtypes (Table S1), heterogeneity in ON history, sex prevalence, and baseline RNFL thickness (Table 1). The limited sample size of the studies may have exacerbated issues related to selection bias and patient heterogeneity, which, despite statistical controls, cannot be fully addressed through methodological adjustments alone.

We failed to detect a significant moderator effect, particularly in the context of optic neuritis history, which aligns with the outcomes derived from our subgroup analyses. However, we should note that the total number of cohorts included in the moderator analysis was fewer than 20 due to the limited number of studies, potentially diminishing the statistical power of these findings.

This study has several limitations. First, due to the availability and quality of published data, potential bias may develop. However, we included as many studies as possible and found no heterogeneity in this meta‐analysis. We aimed to include source studies while ensuring data integrity, with essential details such as OCT device specificity, DMT classification, and time to the active phase of disease. It is therefore possible that not all potentially relevant articles were included. The relatively modest sample size compared with previous systematic reviews may limit the robustness of our conclusions. Second, significant heterogeneity in terms of standard deviation was noted due to incomplete reporting across studies. The statistical methods employed to estimate these missing standard deviations may have introduced bias into our meta‐analysis. Nonetheless, by categorizing DMTs into H‐DMTs and M‐DMTs groups, we have derived novel insights. In addition, the majority of studies included in the M‐DMT group used interferons. Recently approved drugs such as ofatumumab, ponesimod, and ozanimod were included in our search strategy but yielded no results.

In conclusion, our meta‐analysis revealed a protective effect of H‐DMTs on longitudinal thinning of RNFL and GCIPL in patients with RRMS, with a reduced rate of GCIPL degradation compared to those treated with M‐DMTs. Our findings suggest that the GCIPL may be a more appropriate outcome to assess efficacy of DMTs in MS. Further prospective studies and RCTs are needed to refine therapeutic strategies and enhance patient care in MS by addressing the impact of different DMTs on RNFL and GCIPL degeneration.

## Author Contributions

**Xia Zhang:** writing – original draft, investigation, data curation, conceptualization. **Shuang Song:** software, methodology, visualization, formal analysis. **Bo Chen:** data curation, investigation, validation. **Letizia Leocani:** resources, writing – review and editing. **Xinyu Zhao:** methodology, data curation. **Yong Zhong:** writing – review and editing. **Marco Pisa:** conceptualization, writing – original draft, writing – review and editing, supervision, resources. **Srilakshmi M. Sharma:** conceptualization, writing – review and editing, supervision.

## Ethics Statement

The authors have nothing to report.

## Consent

The authors have nothing to report.

## Conflicts of Interest

The authors declare no conflicts of interest.

## Supporting information


Data S1.

Data S2.

## Data Availability

The data supporting the findings of this study are available from the corresponding authors upon reasonable request. Researchers can contact the corresponding authors to request access. Additionally, any Supporting Information related to the manuscript, including statistical analysis scripts and code, will be provided to interested researchers to facilitate replication and further research.
