# Supplementary figures and images for: The Effects of Disease‐Modifying Therapies on Optic Nerve Degeneration in Multiple Sclerosis
Source: Eur J Neurol. 2025 Mar 6;32(3):e70081. doi: 10.1111/ene.70081 (PMC11883414; doi:10.1111/ene.70081)

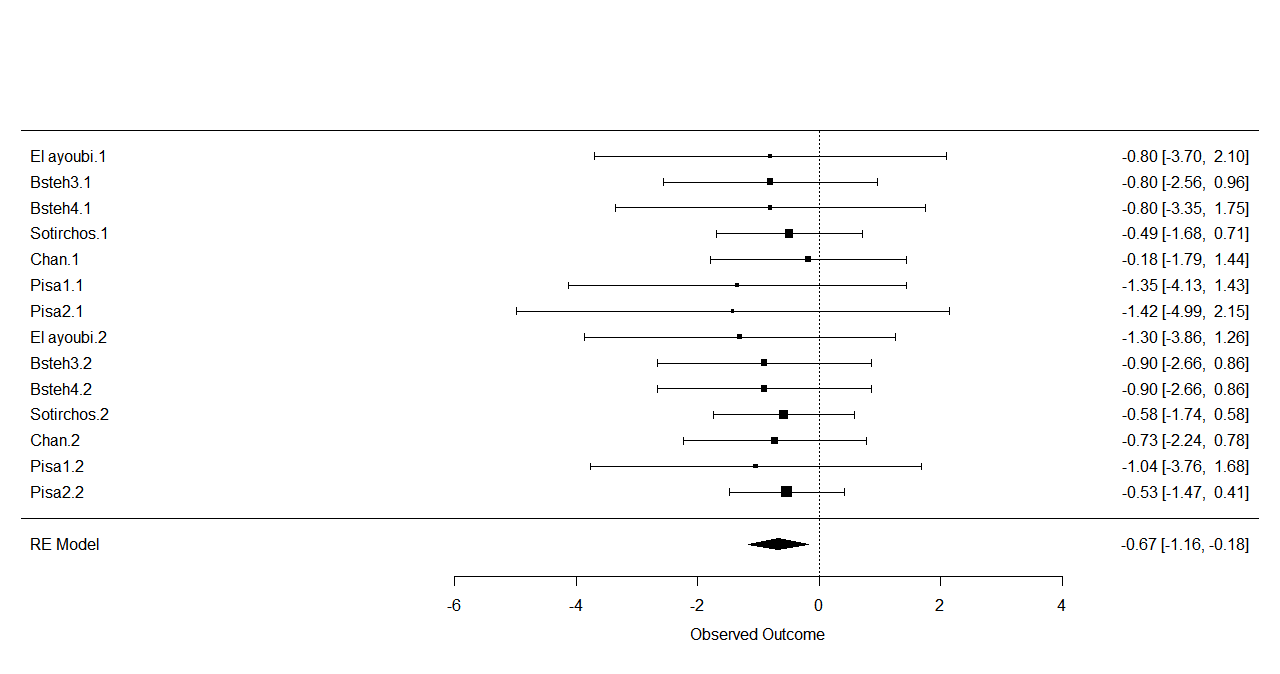

Supplement: Supplementary file 1 — Data S1. [file ENE-32-e70081-s002.tiff]
