# Supplementary material for: The Effects of Disease‐Modifying Therapies on Optic Nerve Degeneration in Multiple Sclerosis
Source: Eur J Neurol. 2025 Mar 6;32(3):e70081. doi: 10.1111/ene.70081 (PMC11883414; doi:10.1111/ene.70081)
Supplement: Supplementary file 2 — Data S2. [file ENE-32-e70081-s001.docx]

**Supplemental Material 1. Search Strategy**

**Search Strategy**

*[ ]: mesh terms available

“Patients”

[Multiple sclerosis]

AND

“Outcomes”

Optic coherence tomography or OCT or RNFL or retinal nerve fibre layer or retinal nerve fiber layer or [Ganglion cell] or ganglion cells or GCC or GCL or GCIPL

Or [visual acuity] or low contrast visual acuity or LCLA or low contrast letter acuity or Sloan letter chart

AND

“Intervention”

Disease modifying therapy or disease modifying therapies or disease modify therapy or disease modify therapies or DMT or disease modifying drug or disease modifying drugs

OR

“Medium efficacy DMT”

Interferon/avonex/interferon-beta/beta-interferon

Peginterferon

Glatiramer acetate/Copaxone/Brabio

Dimethyl fumarate/Tecfidera

Teriflunomide/ Aubagio

OR

“High efficacy DMT”

Fingolimod/ Gilenya

Siponimod/ Mayzent

Ponesimod/ Ponvory

Ozanimod/ Ozanimod

Ocrelizumab/ Ocrevus

Natalizumab/ Tysabri

Rituximab/ Rituxan/MabThera

Daclizumab/ Zinbryta

Alemtuzumab/ Lemtrada

Ofatumumab/ Kesimpta

**Pubmed Search Link:** <https://pubmed.ncbi.nlm.nih.gov/rss/search/1pyhZV_vZSfbTMqM4YmnsByQc8BU_k3wKkkSwYDBmLstgmW9XY/?limit=15&utm_campaign=pubmed-2&fc=20230716111031>

**Search Query:**

("multiple sclerosis"[MeSH Terms] OR "multiple sclerosis"[Title/Abstract]) AND ("optic coherence tomography"[Title/Abstract] OR "OCT"[Title/Abstract] OR "RNFL"[Title/Abstract] OR "retinal nerve fibre layer"[Title/Abstract] OR "retinal nerve fiber layer"[Title/Abstract] OR "ganglion cell"[Title/Abstract] OR "ganglion cells"[Title/Abstract] OR "GCC"[Title/Abstract] OR "GCL"[Title/Abstract] OR "GCIPL"[Title/Abstract] OR "visual acuity"[Title/Abstract] OR "low contrast visual acuity"[Title/Abstract] OR "LCLA"[Title/Abstract] OR "low contrast letter acuity"[Title/Abstract] OR "sloan letter chart"[Title/Abstract] OR "retinal ganglion cells"[MeSH Terms] OR "visual acuity"[MeSH Terms]) AND ("disease modifying therapy"[Title/Abstract] OR "disease modifying therapies"[Title/Abstract] OR "DMT"[Title/Abstract] OR "disease modifying drug"[Title/Abstract] OR "disease modifying drugs"[Title/Abstract] OR "Interferon"[Title/Abstract] OR "Peginterferon"[Title/Abstract] OR "glatiramer acetate"[Title/Abstract] OR "dimethyl fumarate"[Title/Abstract] OR "Teriflunomide"[Title/Abstract] OR "Fingolimod"[Title/Abstract] OR "Siponimod"[Title/Abstract] OR "Ocrelizumab"[Title/Abstract] OR "Natalizumab"[Title/Abstract] OR "Rituximab"[Title/Abstract] OR "Daclizumab"[Title/Abstract] OR "Alemtuzumab"[Title/Abstract] OR "Ofatumumab"[Title/Abstract] OR "Ponesimod"[Title/Abstract] OR "Ozanimod"[Title/Abstract])

**Supplemental Material 2. Data Selection and Extraction**

***Study selection***

After removing duplicate publications, two researchers (X.Z. and B.C.) independently screened the titles and abstracts of the retrieved studies to identify all studies meeting the inclusion criteria. Disagreements during the screening process were resolved by consulting a third expert (M.P.).

The screening revealed that 2 of the 19 included articles shared the same authors and affiliations. The corresponding author (Betsh) of these two articles was contacted for further information.

We have incorporated additional data on delta GCIPL and RNFL thinning from Prof. Leocani's lab, as detailed in two papers[1, 2], categorized by treatment type (M-DMTs vs H-DMTs). This information has been clearly itemized and referenced in supplementary material to ensure comprehensive understanding and transparency.

***Data extraction***

Data were independently extracted from the included studies by two researchers (X.Z. and B.C.) and entered into an Excel spreadsheet. The following data were extracted: first author’s name, publication year, design, group size, patient age, sex ratio, diagnostic criteria, classification of MS, optic neuritis history (extracted per individual), baseline disease activity, baseline EDSS score, OCT device adopted, details of OCT scan mode (ring diameter for RNFL and scan area and units for GCIPL), follow-up durations, and intervention details.

*OCT device compatibility and data conversion*

Only studies that used a Cirrus, Stratus, or Heidelberg Spectralis device for OCT were included. All the authors agreed to include data in the following way based on previous studies [3, 4]. For RNFL thickness, we included data from Cirrus, Stratus, and Spectralis devices, while for GCIPL thickness, only results derived from Cirrus and Spectralis devices were included in the meta-analysis. To ensure comparability, the mean baseline RNFL and GCIPL thicknesses, along with their annual changes, were converted according to a proposed algorithm[5]. Sensitivity analyses were also performed to assess the effect of the device type on OCT measurements to ensure the reliability of this conversion.

*Annualization of RNFL and GCIPL annualization*

Data on annualized changes in the RNLF and GCIPL thickness were extracted directly when they were available. When only baseline and follow-up data were reported, the annualized change was calculated by subtracting the thickness at baseline from the thickness at follow-up and then dividing this value by the follow-up duration. The standard deviation (SD) of the annualized change was derived using the SDs at baseline and follow-up according to the Cochrane Handbook[6]. For studies in which multiple follow-up intervals were reported, the longest duration was selected to calculate the annualized change, thereby reducing test-retest variability.

For studies that reported only the percentage of annualized change, the mean and SD of annualized thickness change were calculated by multiplying the baseline thickness of the mean RNFL or GCIPL by the reported percentage change.

*GCIPL volume conversion*

For the GCIPL analysis, only studies reporting a 6-mm Early Treatment Diabetic Retinopathy Study (ETDRS) circle were included. In studies where the GCIPL volume was reported rather than the thickness, the thickness was calculated as the volume divided by 3*3*π.

*Projected outcomes*

All included studies were screened to determine whether the annualized changes were absolute or projected values. For studies that provided projected values, the authors were contacted to provide the unadjusted data. Two of the 15 included articles provided the projected data. The corresponding author (Wang) and DMT producer (Novartis) were contacted for further information.

*Moderator extraction*

In this research, moderators were systematically extracted from the selected studies. For those studies that reported disease duration, follow-up duration, sex, age, baseline EDSS score, and RNFL or GCIPL thickness separately for different arms or subgroups, individual data points were utilized for analysis. Conversely, for studies that did not provide such detail, the pooled results of each study were applied to both arms or subgroups. Furthermore, the time interval from the active phase was defined as the duration since the most recent optic neuritis attack, as specified in the inclusion criteria, for both eyes.

*Study quality score*

The quality of all included studies was evaluated and rated using a modified version of the Downs and Black checklist[7]. This checklist evaluates three domains – i.e., reporting, external validity, and internal validity – and has a maximum score of 27 points. Two researchers (X.Z. and B.C.) independently rated the studies, and any disagreements were resolved by discussion and consensus with two senior specialists (M.P. and S. Sharma).

In cases of missing data, the corresponding author of the original study was contacted to request access to further study data. Any discrepancies regarding study eligibility and data collection were resolved by discussion or by consulting senior researchers (M.P. and S. Sharma).

**Supplemental Table 1.** Main characteristics of the included studies

| First Author | Publication Year | Study Design | Cohort Size  (Patients/Eyes) | Multiple Sclerosis | Age (mean±SD)# | Female %# | ON History (eye, %)# | Follow-up (yr, mean±SD/median[range])# | OCT device | Disease Modifying Therapy |
| --- | --- | --- | --- | --- | --- | --- | --- | --- | --- | --- |
| Wang[8] | 2022 | RCT | 195/390 | RRMS | 40.85±8.5 | 77.5 | 43.1 | 2 | Stratus | Fingolimod |
| Miscioscia[9]* | 2022 | Prospective observational cohort study | 36/67 | PMS | 51.5±6.0 | 30.6 | 0 | n/a | Spectralis | Ocrelizumab |
| Kilstoner[10]* | 2022 | Prospective observational cohort study | 28/56 | RRMS | 38.1±9.6 | 40.4 | 100 | 6.1±1.4 | Spectralis | Mixed cohort of high efficacy/moderate efficacy/no DMTs |
| Garcia-Martin[11] | 2021 | Prospective observational cohort study | 112/112 | RRMS | n/a | 82.77 | 25.0 | 1 | Cirrus | Fingolimod  /Interferon-β |
| El Ayoubi[12] | 2021 | Prospective observational cohort study | 127/254 | RRMS | 32.9 (18-62.1)/31.2 (18-55.2) | 59.0 | 25.2 | 2.25±1.1 | Cirrus | Fingolimod  /Interferon |
| Sotirchos[13] | 2021 | Prospective observational cohort study | 364/713 | RRMS/PPMS/SPMS | 41.4±9.9/53.3±/51±9.6 | 80.3/55/66.7 | 71.4 | 3.7[2.0-5.9] | Cirrus | Mixed cohort of Natalizumab, rituximab, daclizumab  /interferon, glatiramer acetate |
| Jakimovski[14]* | 2021 | Retrospective study with prospectively collected data | 114/114 | CIS/RRMS | 49.8±12.1 | 77.1 | 59.6 | 5.3±0.8 | Spectralis | Mixed cohort of Natalizumab  /Interferon, glatiramer acetate |
| Lambe[15] | 2021 | Retrospective  observational cohort study | 172/334 | RRMS | 39.4±9.6/37.9±10.2/41.4±9.5 | 76.7 | 31.4 | 1.9(1.5–3.0)/3.0(1.6–4.7)/2.7 (2.3–3.8) | Cirrus | Rituximab, Natalizumab  /Glatiramer Acetate |
| Klumbies.[16] | 2021 | RCT | 16/31 | PMS | 50.7±6.9 | 43.8 | n/a | 2 | Spectralis | Placebo |
| Bsteh[17] | 2020 | Prospective observational cohort study | 168/168 | RRMS | 34.8±9.2 | 71.4 | 20.8 | 3 | Spectralis | Natalizumab, fingolimod, alemtuzumab, cladribine/interferon, glatiramer acetate, dimethylfumarate, teriflumomide |
| Chan[18] | 2020 | Retrospective study with prospectively collected double cohort data | 45/88 | RRMS | 35.05±8.69 | 84.4 | 38.8 | 5±1.5/5 | Spectralis | Alemtuzumab  /Interferon, Glatiramer Acetate |
| Pisa[2] | 2020 | Prospective observational cohort study | 43/86 | RRMS/SPMS/PPMS | 46.2±13.3 | 74.1 | 61.1 | 2.43±1.1 | Spectralis | Natalizumab, Fingolimod/Interferon, Azathioprine Glatiramer Acetate |
| Bermel[19] | 2020 | RCT | 123/246 | PMS | 56.89±6.49 | 57 | n/a | 2 | Cirrus/Spectralis | Placebo |
| Bsteh.[20] | 2019 | Prospective observational cohort study | 141/141 | RRMS | 35±9.4 | 75.9 | 20.6 | 3 | Spectralis | Natalizumab, fingolimod, alemtuzumab/interferon, glatiramer acetate, dimethylfumarate, teriflumomide |
| Zivadinov[21] | 2018 | Prospective observational cohort study | 60/120 | RRMS | 42.7±10.2 | 75.0 | 43.3 | 2 | Spectralis | Glatiramer Acetate |
| Talmage[22] | 2017 | Open label clinical trial | 15/30 | RRMS | 39±9 | 86.7 | n/a | 2 | Spectralis | Natalizumab |
| Pisa[23] | 2017 | Prospective observational cohort study | 72/106 | RRMS/SPMS/PPMS | 37±9.6 | 61.1 | 23.9 | 2.00±0.54 | Spectralis | Natalizumab, Fingolimod/Interferon, Azathioprine Glatiramer Acetate |
| Button[24] | 2017 | Retrospective study | 157/308 | RRMS | 41.6±9.6/40.5±11.1/45.0±9.0/39.4±10.6 | 81/86/75/74 | 52/49/52/52 | 3.2±1.3/3.0±1.5/2.9±1.5/3.0±1.4 | Cirrus | Natalizumab, Fingolimod/Interferon |
| Winges[25]* | 2017 | Prospective Observational cohort study | 47/80 | PMS | 59±6.4 | 59.6 | 34 | 2 | Cirrus | 45% on DMT (Glatiramer Acetate/Interferon) |
| Garcia-Martin[26]* | 2017 | Prospective observational cohort study | 102/200 | RRMS | 41.12±11.45 | 68 | 25.5 | 5 | Cirrus | 62% on DMT (Glatiramer Acetate/Interferon) |
| Waubant[27] | 2013 | RCT | 21/42 | RRMS/CIS | 32.4±7.85 | 66.7 | n/a | 2 | Stratus | Interferon+Placebo |

* five additional articles with mixed DMT administration were added to the subgroup analysis.

# Age, sex, ON history, and follow-up duration were reported as a single number for each study if presented in this way. For studies that did not provide pooled data, these details were reported separately for each group within the study.

Abbreviations: SD: standard deviation; ON: optic neuritis; OCT: optic coherence tomography; DMT: disease-modifying therapies; M-DMTs: moderate efficacy DMTs; H-DMT: high efficacy DMTs; RRMS: relapsing-remitting multiple sclerosis PPMS: primary progressive multiple sclerosis; SPMS: secondary progressive multiple sclerosis; RCT: randomized controlled trials; n/a: not available.

Figure Legend of Supplemental Figure 1:

In this sensitivity analysis, a significant annualized RNFL thinning was observed between baseline and follow-up OCT assessments across the study included, with an effect size of -0.6706 (95% CI=[-1.15 to -0.18], *p=*0.0107). Specifically, the M-DMT group showed a significant decrease in RNFL thickness between baseline and follow-up, with a small to moderate effect size (gM-DMTs = -0.6991, 95% CI = [-1.32 – -0.08], *p=*0.0243), while no significant decrease in RNFL thickness was detected in the H-DMT group (gH-DMTs = -0.6215, 95% CI = [-1.43–0.19], *p=*0.1062). However, the difference in annualized RNFL thinning between the H-DMTs and M-DMTs cohorts did not reach statistical significance (*p=*0.87). The SD between outcomes (τo) was <0.0001, indicating minimal variance attributable to heterogeneity across the included studies.

**REFERENCES CITED IN SUPPLEMENTARY MATERIALS**

1. Pisa, M., et al., *No evidence of disease activity is associated with reduced rate of axonal retinal atrophy in MS.* Neurology, 2017. **89**(24): p. 2469-2475.

2. Pisa, M., et al., *Subclinical neurodegeneration in multiple sclerosis and neuromyelitis optica spectrum disorder revealed by optical coherence tomography.* Mult Scler, 2020. **26**(10): p. 1197-1206.

3. Jeoung, J.W. and K.H. Park, *Comparison of Cirrus OCT and Stratus OCT on the ability to detect localized retinal nerve fiber layer defects in preperimetric glaucoma.* Invest Ophthalmol Vis Sci, 2010. **51**(2): p. 938-45.

4. Brennen, P.M., L. Kagemann, and T.R. Friberg, *Comparison of StratusOCT and Cirrus HD-OCT imaging in macular diseases.* Ophthalmic Surg Lasers Imaging, 2009. **40**(1): p. 25-31.

5. Kenney, R., et al., *Normative Data and Conversion Equation for Spectral-Domain Optical Coherence Tomography in an International Healthy Control Cohort.* J Neuroophthalmol, 2022. **42**(4): p. 442-453.

6. Tran, K.A., et al., *Characterization of the Clinical Evidence Supporting Repository Corticotropin Injection for FDA-Approved Indications: A Scoping Review.* JAMA Intern Med, 2022. **182**(2): p. 206-217.

7. Downs, S.H. and N. Black, *The feasibility of creating a checklist for the assessment of the methodological quality both of randomised and non-randomised studies of health care interventions.* J Epidemiol Community Health, 1998. **52**(6): p. 377-84.

8. Wang, L., et al., *Baseline retinal nerve fiber layer thickness as a predictor of multiple sclerosis progression: new insights from the FREEDOMS II study.* Eur J Neurol, 2023. **30**(2): p. 443–452.

9. Miscioscia, A., et al., *Retinal inner nuclear layer thinning is decreased and associates with the clinical outcome in ocrelizumab-treated primary progressive multiple sclerosis.* J Neurol, 2022. **269**(10): p. 5436-5442.

10. Klistorner, A., et al., *Long-term effect of permanent demyelination on axonal survival in multiple sclerosis.* Neurol Neuroimmunol Neuroinflamm, 2022. **9**(3): p. e1155.

11. Garcia-Martin, E., et al., *Progressive functional and neuroretinal affectation in patients with multiple sclerosis treated with fingolimod.* J Neuroophthalmol, 2021. **41**(4): p. e415–e423.

12. El Ayoubi, N.K., et al., *Effect of fingolimod vs interferon treatment on OCT measurements and cognitive function in RRMS.* Mult Scler Relat Disord, 2021. **53**: p. 103041.

13. Sotirchos, E.S., et al., *Progressive multiple sclerosis is associated with faster and specific retinal layer atrophy.* Ann Neurol, 2020. **87**(6): p. 885–896.

14. Jakimovski, D., et al., *Clinical effects associated with five-year retinal nerve fiber layer thinning in multiple sclerosis.* J Neurol Sci, 2021. **427**: p. 117552.

15. Lambe, J., et al., *Modulation of retinal atrophy with rituximab in multiple sclerosis.* Neurology, 2021. **96**(20): p. e2525–e2533.

16. Klumbies, K., et al., *Retinal Thickness Analysis in Progressive Multiple Sclerosis Patients Treated With Epigallocatechin Gallate: Optical Coherence Tomography Results From the SUPREMES Study.* Front Neurol, 2021. **12**: p. 615790.

17. Bsteh, G., et al., *Macular ganglion cell-inner plexiform layer thinning as a biomarker of disability progression in relapsing multiple sclerosis.* Mult Scler, 2021. **27**(5): p. 684-694.

18. Chan, J.K., et al., *Long-term stability of neuroaxonal structure in alemtuzumab-treated relapsing-remitting multiple sclerosis patients.* J Neuroophthalmol, 2020. **40**(1): p. 37–43.

19. Bermel, R.A., et al., *Optical coherence tomography outcomes from SPRINT-MS, a multicenter, randomized, double-blind trial of ibudilast in progressive multiple sclerosis.* Mult Scler, 2021. **27**(9): p. 1384-1390.

20. Bsteh, G., et al., *Peripapillary retinal nerve fibre layer thinning rate as a biomarker discriminating stable and progressing relapsing-remitting multiple sclerosis.* Eur J Neurol, 2019. **26**(6): p. 865-871.

21. Zivadinov, R., et al., *The Effect of Glatiramer Acetate on Retinal Nerve Fiber Layer Thickness in Patients with Relapsing-Remitting Multiple Sclerosis: A Longitudinal Optical Coherence Tomography Study.* CNS Drugs, 2018. **32**(8): p. 763-770.

22. Talmage, G.D., et al., *Natalizumab stabilizes physical, cognitive, MRI, and OCT markers of disease activity: a prospective, non-randomized pilot study.* PLoS One, 2017. **12**(4): p. e0173299.

23. Pisa, M., et al., *No evidence of disease activity is associated with reduced rate of axonal retinal atrophy in MS.* Neurology, 2017. **89**(24): p. 2469–2475.

24. Button, J., et al., *Disease-modifying therapies modulate retinal atrophy in multiple sclerosis: a retrospective study.* Neurology, 2017. **88**(6): p. 525–532.

25. Winges, K.M., et al., *Longitudinal optical coherence tomography study of optic atrophy in secondary progressive multiple sclerosis: Results from a clinical trial cohort.* Mult Scler, 2019. **25**(1): p. 55-62.

26. Garcia-Martin, E., et al., *Retinal and Optic Nerve Degeneration in Patients with Multiple Sclerosis Followed up for 5 Years.* Ophthalmology, 2017. **124**(5): p. 688-696.

27. Waubant, E., et al., *A randomized controlled phase II trial of riluzole in early multiple sclerosis.* Ann Clin Transl Neurol, 2014. **1**(5): p. 340-7.
